# Overcoming barriers to cervical screening attendance among underrepresented populations in Europe

**DOI:** 10.1016/j.lanepe.2024.100932

**Published:** 2024-05-16

**Authors:** Sophie Mulcahy Symmons, Amanda Drury, Aoife De Brún

**Affiliations:** a School of Nursing, Midwifery and Health Systems, University College Dublin, Ireland; b UCD Centre for Interdisciplinary Research, Education, and Innovation in Health Systems (UCD IRIS Centre), School of Nursing, Midwifery and Health Systems, University College Dublin, Ireland; c School of Nursing, Psychotherapy and Community Health, Dublin City University, Dublin 9, Glasnevin, Ireland

The World Health Organisation (WHO) European region released a roadmap in response to the call to action to eliminate cervical cancer by the end of the century with the core principle of “leaving no one behind”.^1^ To eliminate cervical cancer (reduce incidence below 4 in 100,000) equitably, high rates of HPV vaccination, screening participation and treatment of pre-cancers and management of invasive cancers must be achieved.

In Europe there were 58,129 new cases of cervical cancer and 26,950 deaths from cervical cancer in 2022.^2^ The average age-standardised incidence of cervical cancer in Europe is 10.6 per 100,000 cases per year. However, some countries have a higher burden of cervical cancer, for instance, in Romania the incidence is 21.7 per 100,000.^2^ Many European countries announced steps towards the elimination of cervical cancer. Sweden is on track to eliminate cervical cancer before 2030, while The UK and Ireland both announced their projected date as 2040.

Cervical screening to promote early detection is an important component in the strategy to eliminate cervical cancer. A key step in delivering cervical screening is recognising which populations are less likely to attend screening. The social determinants of health have a significant impact on risk of underrepresentation in screening; determinants include low socioeconomic position (including low education and income levels), place of residence, ethnicity, migrant status, older age, having a disability, identifying as LGBTQI+, experience of sexual abuse and drug use.^3^, ^4^, ^5^

According to the 2015 European Health Interview Survey, women in Eastern Europe were less likely to have cervical screening than those in Western Europe. Furthermore, women born outside of the EU, with low educational level and low household income, were less likely to have had cervical screening in the past three years than native-born women, women with high education and high household income.^6^ These data highlight some of the many inequalities in screening and must not be ignored when planning the path to eliminating cervical cancer.^7^

Many barriers exist that impact participation in screening (Fig. 1). Individuals may face a combination of barriers such as bureaucracy in the health system impacting registration and invitation, financial barriers, discrimination, negative experiences, access issues, lack of appropriate information or cultural beliefs, fear, conflicting priorities and low awareness.^3^^,^^8^ In order to break down these barriers, it is necessary to understand them from a multi-level point of view. We propose an intersectional and person-centred approach be adopted that considers individual, service and system level barriers, to achieve equitable provision and access to cervical screening.

**Fig. 1:**
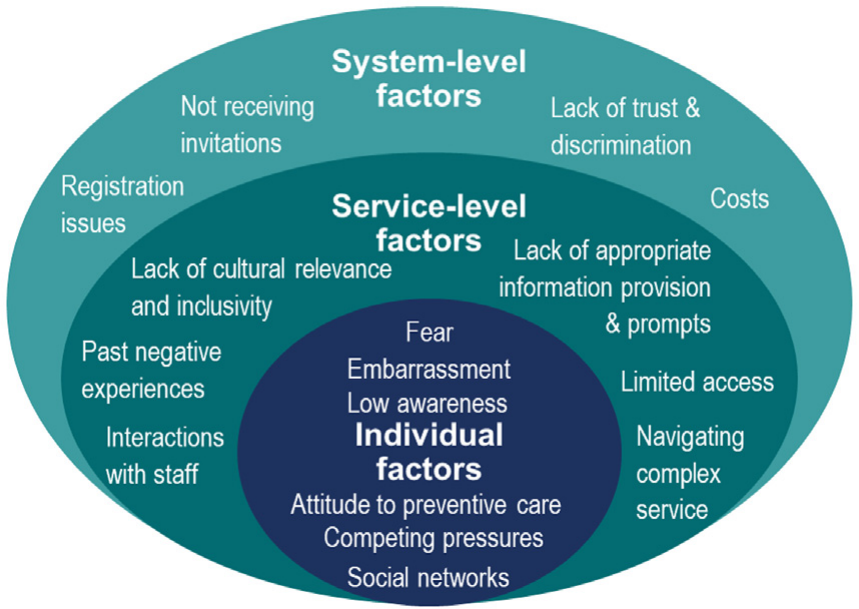
System-, service- and individual-level barriers to screening among underrepresented populations in Europe.

Sources.^3^^,^^8^

Core to promotion of screening is first understanding individuals' awareness, fear, embarrassment, and social and cultural norms. Our view is that evidence-based interventions should be co-designed and delivered to increase participation in a way that is meaningful to the population. Collaborating with communities at risk of underrepresentation in screening will develop trust and unveil barriers, needs and possible solutions to increase accessibility and acceptability of screening, such as developing faith-based health education for Muslim women.^7^^,^^9^

Typical practices to reduce service-level barriers include sending invitations and reminders, providing the service close one’s residence (or taking the service to service users), flexible appointments times, support with navigation through service, and access to services with female staff.^5^ We see healthcare professionals (HCPs) playing a key role in supporting participation through their rapport with patients. HCPs should share trusted and clear communication materials about cervical cancer and screening.^1^ Services should be welcoming and inclusive to all communities, encompassing provision of information in accessible and acceptable formats and HCP training to initiate dialogues that acknowledge barriers to participation.

Regarding system-level barriers, countries should develop policies that focus on reducing inequities in screening participation. However, currently only six in twenty-two European countries have policies to promote cervical screening among vulnerable populations, and few monitor their engagement with screening.^5^ Health systems can consider updating registration and invitation systems to ensure individuals are not lost at the first point of contact. Screening and follow-up care should be provided for free supporting those with limited resources.^7^ Finally, self-sampling is a promising alternative to clinician-collected samples as studies show it increases uptake of screening and is acceptable among women and could be evaluated for acceptability and cost effectiveness across Europe.^10^

Actions to increase participation in cervical screening must be in partnership with populations at risk of underrepresentation in screening. By first taking time to build trust with populations and understand the multi-level barriers they encounter, it will lead to identifying and prioritising their needs and collaboratively designing appropriate solutions to provision of screening. Recognition of system, service and individual level barriers and meaningful collaboration with all stakeholders will enable an equitable path to eliminating cervical cancer.

## Contributors

SMS–Conceptualisation, and writing: original draft, review and editing. AD–writing: review and editing. ADB–writing: review and editing.

## Declaration of interests

The authors have no competing interests.
